# Longitudinal associations among physical activity and sitting with endocrine symptoms and quality of life in breast cancer survivors: A latent growth curve analysis

**DOI:** 10.1002/cam4.6581

**Published:** 2023-09-28

**Authors:** Alexander R. Lucas, Youngdeok Kim, Autumn Lanoye, R. Lee Franco, Arnethea L. Sutton, Jessica G. LaRose, Masey Ross, Vanessa B. Sheppard

**Affiliations:** ^1^ Department of Health Behavior and Policy Virginia Commonwealth University Richmond Virginia USA; ^2^ Department of Internal Medicine – Cardiology Virginia Commonwealth University Richmond Virginia USA; ^3^ Department of Kinesiology and Health Sciences Virginia Commonwealth University College of Humanities and Sciences Richmond Virginia USA; ^4^ Massey Cancer Center Virginia Commonwealth University Richmond Virginia USA; ^5^ Department of Internal Medicine‐ Oncology Virginia Commonwealth University Health System Richmond Virginia USA; ^6^ Office of Health Equity and Disparities Research, Massey Cancer Center Virginia Commonwealth University Richmond Virginia USA

**Keywords:** breast cancer, lifestyle, physical activity, survivorship, symptoms

## Abstract

**Purpose:**

Adjuvant endocrine therapy (AET) often causes debilitating endocrine symptoms that compromise quality of life (QOL) in women diagnosed with hormone receptor positive breast cancer (BC). We examined whether greater levels of physical activity (PA) or prolonged sitting were associated with reduced side effects or worse side effects of AET, respectively.

**Methods:**

We used parallel process latent growth curve models to examine longitudinal patterns in PA and sitting behaviors, and their association with endocrine symptoms and QOL over 3 years of follow‐up in 554 female BC survivors undergoing AET.

**Results:**

At baseline, women were a mean age of 59 years, mostly white (72%), with overweight/obesity (67%), and approximately 50% were within 1 year of diagnosis. Unconditional models showed significant increases in PA (*p* < 0.01) over time but no change in sitting. Endocrine symptoms, general and BC‐specific QOL all significantly worsened over time (*p* < 0.01). Parallel process models showed no cross‐sectional or longitudinal associations between PA and endocrine symptoms. Higher levels of baseline PA were associated with higher baseline QOL scores (*p* = 0.01) but changes in PA were not associated with changes in QOL. Conversely, more sitting at baseline was associated with worse endocrine symptoms, general and BC specific QOL (*p*s <0.01). At baseline, having better QOL scores was associated with increases in sitting (*p*s <0.01), while having worse endocrine symptoms was associated with a slower rate of increase in sitting (*p* < 0.01). Increases in sitting time were also associated with a slower rate of increase in endocrine symptoms (*p* = 0.017). Model fit statistics (x2, CFI, TLI, SRMR) were acceptable.

**Conclusion:**

Both PA and sitting behaviors are important for the management of symptoms and in maintaining QOL in BC survivors. Women with already high symptom burden do not increase sitting time further but having better general and BC specific QOL to begin with means a greater decline over time.

## Graphical abstract

Adjuvant endocrine therapy (AET) often causes debilitating endocrine symptoms that compromise quality of life (QOL) in women diagnosed with hormone receptor positive breast cancer (BC). We used parallel process latent growth curve models to examine longitudinal patterns in PA and sitting behaviors, and their association with endocrine symptoms and QOL over 3 years of follow‐up in 554 female BC survivors undergoing AET. Parallel process models showed no cross‐sectional or longitudinal associations between PA and endocrine symptoms.

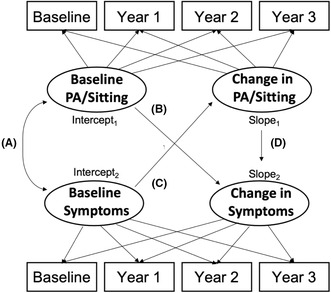

## 1 INTRODUCTION

Early detection and effective treatments have increased survival rates for women diagnosed with breast cancer (BC),^1^ with adjuvant endocrine therapy (AET) being an important option for women diagnosed with hormone receptor positive (HR+) BC.^2^ Yet, AET can also frequently produce negative side effects such as joint pain and stiffness,^3^ loss of bone mineral density,^4^ and vasomotor and gynecological symptoms that compromise quality of life (QOL). Unfortunately, these symptoms often lead to poor adherence with up to 50% of women either not initiating or completing their recommended 5‐year therapy regimens.^5^, ^6^

To optimize their health status, BC survivors should therefore aim to adopt or maintain healthy lifestyle behaviors that include a healthy diet and physical activity.^7^ BC survivors who gain >5% body weight post‐diagnosis have a 12% higher all‐cause mortality relative to BC survivors who maintain their weight.^8^ Weight gain is also strongly associated with the onset of metabolic dysfunction and cardiovascular disease^9^ and may be an important factor impacting symptoms associated with AET. Current guidelines recommend at least 150 min of moderate to vigorous physical activity (MVPA) or 75 min of VPA per week while also avoiding inactivity.^10^, ^11^ Engaging in PA is also an important part of weight management post BC therapy and may be able to mitigate many of the side effects of AET^12^, ^13^; however, higher intensity PA may also be challenging^14^ due to the impact of BC‐treatments on functional capacity and health‐related QOL. It should also be recognized that socio‐demographic factors and pre‐existing comorbidities may make achieving recommended levels of PA difficult.^15^ Consequently, independent of PA, BC survivors should aim to reduce sedentary behaviors (e.g., sitting) as a means of increasing light intensity PA that is also important for weight management,^16^ metabolic and cardiovascular health, and managing symptoms such as fatigue.^17^ What is not yet known is how patterns of PA, sitting and endocrine symptoms change over time following the initiation and adherence to AET.

A small number of previous randomized controlled studies have examined the effects of either aerobic or resistance training (or both) on endocrine therapy‐related symptoms such as arthralgias, hot flushes, bone loss and QOL generally.^18^, ^19^, ^20^, ^21^ Findings from these studies has been somewhat mixed with some showing no significant benefits of exercise training over non‐exercising controls on endocrine symptoms^19^, ^20^ while others find only certain domain‐specific benefits^21^, ^22^ or improved general QOL. Furthermore, metanalytic data from nine trials with 743 participants across multiple countries has shown specific musculoskeletal benefits of exercise, with improvements in pain, stiffness, and grip strength.^23^ However, due the heterogeneity in: (i) the measures used to assess endocrine symptoms, (ii) in the individual study samples (e.g., small sample sizes, differential enrollment relative to treatment completion), and (iii) a lack of focus on sedentary behaviors, we sought to further examine these associations in the current observational study among women not specifically being asked to exercise or change their activity behaviors.

Disparities in adherence to AET have been noted among insured BC survivors by age and race^24^ and while previous studies have examined PA patterns in BC patients following treatment,^25^ they do not include representative numbers of Black women. Furthermore, few studies have examined cross‐sectional and longitudinal associations among PA and sitting with general and specific aspects of QOL among women actively undergoing AET. The Women's Hormonal Initiation and Persistence (WHIP) study is an observational cohort study with up to 3 years of follow‐up data on a diverse group of women that allows for the examination of these questions given its inclusion of clinical‐ and treatment‐related data among BC survivors.^26^ We hypothesized that at baseline, higher levels of self‐reported PA would be associated with better QOL and fewer endocrine symptoms. Further, we hypothesized that PA would increase over time as women recovered from BC treatment and that increases in PA would be associated with improving QOL and reduced endocrine symptoms. With regards to sedentary sitting time, we also hypothesized both cross‐sectional and longitudinal associations with QOL. Specifically, more sitting time at baseline would be associated with worse QOL and endocrine symptoms while increased sitting time would be associated with worsening QOL and symptoms. We utilize a rigorous analytical method, parallel latent growth curve modeling (LGCM), to estimate (1) average changes in PA, sitting, and QOL and endocrine symptoms; and (2) the extent to which changes in PA and sitting explain the change in general and specific aspects of QOL over a 3‐year period, while controlling for time‐varying confounding effects of the respective behaviors.

## 2 MATERIALS AND METHODS

The current study is a secondary analysis of the Women's Hormonal Initiation and Persistence (WHIP) study. WHIP was a longitudinal observational cohort study designed to examine patterns of adherence to AET (R01CA154848). All study procedures were approved by Institutional Review Boards at participating sites. The study design, recruitment and data collection methods have previously been described in detail.^26^ Briefly, women diagnosed with BC were identified across two primary geographic regions (Midwest, Southeast) from academic medical centers, from integrated health systems, and via community outreach efforts. Eligibility criteria included having a diagnosis of HR+ BC (confirmed via laboratory reports), filling a script for AET within 1 year after diagnosis and within 3 months of the baseline, being 21 years old or older, having initiated AET, and being able to speak English or Spanish. Women were deemed potentially eligible on the basis of cancer registries, and via pharmacy records. They were then mailed letters that described the study protocol and were asked to call a toll‐free number or to return a stamped self‐addressed postcard if they did not want to be contacted about the study. Women recruited via community outreach were screened for eligibility by a clinical research assistant. Research assistants then contacted women who did not opt out within a 2‐week time frame, confirmed the level of interest, consented interested participants, and scheduled phone interviews to complete the survey. Data were collected via standardized surveys that were administered according to a woman's preference either via telephone by a trained clinical research assistant or via a secure online survey (REDCap). The baseline cohort included 594 women, with a decreasing number of valid samples across the 3 years of follow‐up visits (missing n at Year 1 = 162, Year 2 = 220, and Year 3 = 405). Women were excluded from the analysis if they had invalid and/or missing data for study outcomes across all years (*n* = 40), leaving a valid sample of 554 for longitudinal analysis.

### 2.1 Measures

PA and sitting time were measured with the short form of the International Physical Activity Questionnaire (IPAQ).^27^, ^28^ Participants were asked to self‐report frequency and duration of walking, or participating in moderate‐intensity activities (e.g., carrying light loads and bicycling at a regular pace) and vigorous‐intensity activities (heavy lifting and aerobics) during the past 7 days. IPAQ data were cleaned by following the rules set by the IPAQ executive committee^29^ including the exclusion of unreasonably high levels of PA data (e.g., total PA exceeding 16 h per day) and truncation of the self‐reported duration of each type of PA when exceeding 4 h per day. Metabolic equivalents of task (METs) of 3.3, 4.0, and 8.0 were subsequently applied to walking, moderate‐ and vigorous‐intensity activities, respectively, to calculate total MET‐hours per week. As part of IPAQ, participants reported the average daily time spent in sitting during the past 7 days.

QOL was measured with the Functional Assessment of Cancer Therapy (FACT‐General) questionnaire, as well as with the additional Breast Cancer (FACT‐B), and Endocrine Symptoms (FACT‐ES) subscales, disease‐ and symptom‐specific subscales, respectively. QOL scores (FACT‐G, FACT‐B) and ES‐subscale only scores were all assessed as continuous variables and examined for their relationship with PA and sitting. Higher FACT‐G and FACT‐B scores indicate better health‐related QOL and higher ES‐subscale scores indicate worse adjuvant endocrine therapy‐related symptoms. Previous studies have reported minimally important differences of 5–6 points for the FACT‐G and 2–3 points for FACT‐B subscale among breast cancer survivors.^30^ In the present study, reliability of each measure was acceptable with a Cronbach's alpha ranging between 0.87 and 0.91 (FACT‐G), between 0.89 and 0.92 (FACT‐B), and between 0.78 and 0.80 (ES‐subscale scores) across the years. Baseline sociodemographic characteristics were obtained via survey while clinical variables were confirmed via medical records. Sociodemographic variables included age, self‐identified race (Black vs. white), marital status (married/partnered vs. others), education (high school or lower vs. college or higher), employment (yes vs. no), household income (<$60k, $60k through <$100k, $100k thru <$150k and ≥$150k). Clinical variables included BMI (<25 kg/m^2^, 25 through 30 kg/m^2^, and ≥30 kg/m^2^), time since diagnosis (<1 year vs. ≥1 year), BC stage at diagnosis (Stage I, II, or III), human epidermal growth factor receptor 2 (HER2) status (negative or positive), time since AET initiation (<6 months vs. ≥6 months), surgery type (mastectomy vs. lumpectomy), receipt of chemotherapy (yes vs. no), and receipt of radiation (yes vs. no).

### 2.2 Statistical analyses

Descriptive statistics of the baseline characteristics were calculated and compared by the number of valid visits (i.e., 1–2 vs. 3–4 valid visits) to examine potential baseline characteristics associated with missingness of the study variables (i.e., PA, sitting, QOL, and endocrine symptoms). Descriptive statistics of the study variables were calculated across the visits, and linear trends over time were estimated using an orthogonal polynomial contrast in a linear mixed model with an unstructured covariance structure for repeated measures.

**FIGURE 1:**
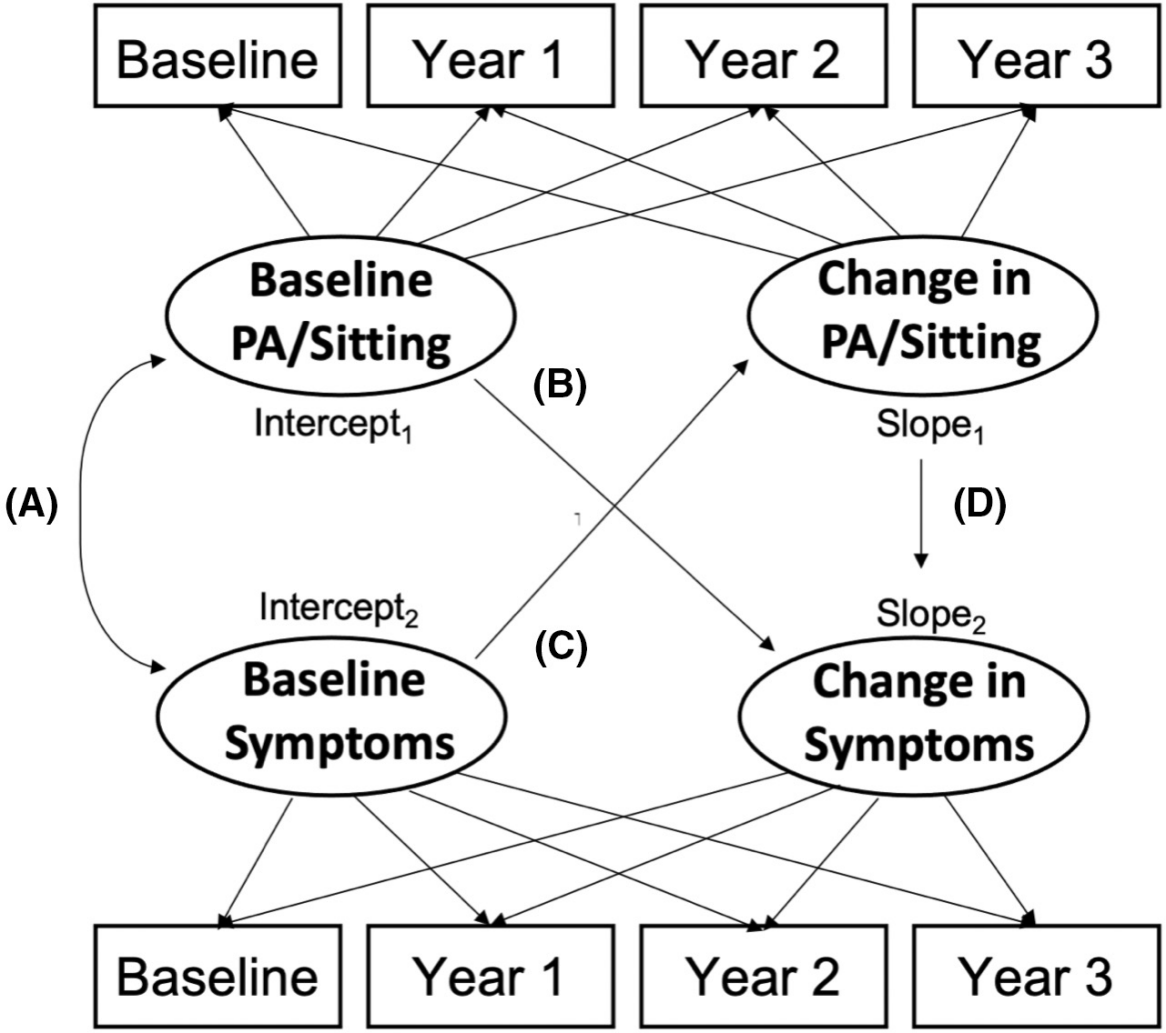
Parallel process latent growth curve model. (A) Intercept‐to‐Intercept correlation (i.e., cross‐sectional association between the two study variables at baseline); (B,C) Intercept‐to‐Slope coefficient (i.e., the prospective association of the baseline level of one variable with a rate of change in the other variable); and (D) Slope‐to‐Slope coefficient (i.e., the unidirectional association of the rate of change of PA or Sitting time with the rate of change of the endocrine symptoms and QOL over years).

A series of latent growth curve models (LGCM) were examined for the primary analyses. First, an unconditional LGCM was established for each study variable to examine longitudinal trajectories over time. Three latent growth factors, including intercept (i.e., baseline level), slope (i.e., rate of change per year), and correlation between intercept and slope, were estimated from each model. We then examined the longitudinal associations of PA and sitting with QOL and endocrine symptoms using the parallel process LGCM. Six parallel process LGCMs were tested for each pair of PA or sitting and FACT‐ES subscale, FACT‐G and FACT‐B variables. Each of the parallel LGCM's was adjusted for age (years), self‐identified race (Black vs. white) and the time since initiation of AET, except for the model examining the association of sitting time with the FACT‐B, where the models were adjusted for age and race only, due to a model convergence issue with the time since AET initiation. A schematic diagram of the parallel process LGCM is presented in Figure 1. The primary parameters of interest included: (a) Intercept‐to‐Intercept correlation (i.e., cross‐sectional association between the two study variables at baseline); (b) and (c) Intercept‐to‐Slope coefficient (i.e., the prospective association of the baseline level of one variable with a rate of change in the other variable); and (d) Slope‐to‐Slope coefficient (i.e., the unidirectional association of the rate of change of PA or Sitting time with the rate of change of the endocrine symptoms and QOL over years). The χ2 statistic assessed absolute fit of the model to the data. The model‐data‐fit of the LGCMs was also assessed based on the comparative fit index (CFI), Tucker–Lewis index (TLI), and root mean square error of approximation (RMSEA). The model was considered acceptable if the CFI and TLI ≥0.90 and RMSEA<0.10^31^, ^32^; yet, less emphasis was given to the RMSEA, particularly when evaluating the unconditional LGCM with few degrees of freedom.^33^ The modification indices were also assessed to improve the model‐data fit with a consideration of theoretical justification and interpretability. The LGCM analyses were examined using full‐information maximum likelihood estimator accounting for missing data under the assumption of at least missing at random. We also conducted a follow‐up sensitivity analysis by excluding individuals with two or more missing study variables and compared the results. SAS v9.4 (SAS Institute) was used for data management and the Mplus v7.2 (Muthén & Muthén) was used for LGCM analyses.

## 3 RESULTS

### 3.1 Sample characteristics

The current analyses included 554 women who had complete PA, sitting and QOL data at baseline visit. Table 1 details the participant characteristics by the number of follow up visits where data contributed to longitudinal analyses. At baseline, women enrolled in the WHIP study were a mean age of 58.9 years, majority white (72%), mostly married or partnered (65%), generally having a college education or higher (86%). Approximately half of women in our cohort were less than 1 year from their BC diagnosis having started AET within the past 6 months. We found differences in the years of follow‐up data available by race, education, and household income such that Black women, women with less than a college education and those with the lowest income bracket were less likely to contribute 2–3 years of data versus 1–2 years.

**TABLE 1:** Baseline characteristics of the breast cancer survivors of the WHIP study by the number of valid data points over 4 years.

	Total, *n* (%)	Number of valid data points (max: 4 years)^a^		*p*‐value^b^
		1–2 years	2–3 years	
	554 (100%)	273 (49.28%)	281 (50.72%)	
Age (years)	58.93 ± 10.96	58.96 ± 12.14	58.90 ± 9.70	0.949
Race				**<0.001**
Black	157 (28.34%)	103 (37.73%)	54 (19.22%)	
White	397 (71.66%)	170 (62.27%)	227 (80.78%)	
Marital status				0.414
Married/partnered	359 (64.92%)	172 (63.24%)	187 (66.55%)	
Others	194 (35.08%)	100 (36.76%)	94 (33.45%)	
Unknown/missing	1	1	0	
Education				**0.018**
High school or lower	76 (13.87%)	47 (17.41%)	29 (10.43%)	
College or higher	472 (86.13%)	223 (82.59%)	249 (89.57%)	
Unknown/missing	6	3	3	
Employment status^c^				0.532
Yes (employed)	310 (58.82%)	150 (57.47%)	160 (60.15%)	
No (unemployed)	217 (41.18%)	111 (42.53%)	106 (39.85%)	
Unknown/missing	27	12	15	
Household income				**0.023**
<$60k	162 (31.03%)	94 (36.72%)	68 (25.56%)	
$60k through <$100	153 (29.31%)	63 (24.61%)	90 (33.83%)	
$100k through <$150k	104 (19.92%)	52 (20.31%)	52 (19.55%)	
≥$150 k	103 (19.73%)	47 (18.36%)	56 (21.05%)	
Unknown/missing	32	17	15	
Time since diagnosis				0.276
<1 year	266 (53.63%)	131 (56.22%)	135 (51.33%)	
≥1 year	230 (46.37%)	102 (43.78%)	128 (48.67%)	
Unknown/missing	58	40	18	
Stage at diagnosis				0.069
I	292 (60.71%)	136 (57.63%)	156 (63.67%)	
II	148 (30.77%)	73 (30.93%)	75 (30.61%)	
III	41 (8.52%)	27 (11.44%)	14 (5.71%)	
Unknown/missing	73	37	36	
HER2 status				0.276
Negative	242 (92.02%)	106 (89.07%)	136 (94.44%)	
Positive	21 (7.98%)	13 (10.92%)	8 (5.55%)	
Unknown/missing	291	154	137	
Time since AET initiation				0.621
<6 months	234 (49.26%)	116 (50.43%)	118 (48.16%)	
≥6 months	241 (50.74%)	114 (49.57%)	127 (51.84%)	
Unknown/missing	79	43	36	
Chemotherapy				0.600
Yes	204 (39.38%)	99 (40.57%)	105 (38.32%)	
No	314 (60.62%)	145 (59.43%)	169 (61.68%)	
Unknown/missing	36	29	7	
Radiation treatment				0.392
Yes	329 (67.14%)	152 (65.24%)	177 (68.87%)	
No	161 (32.86%)	81 (34.76%)	80 (31.13%)	
Unknown/missing	64	40	24	
Surgery type^d^				0.416
Mastectomy	175 (44.76%)	85 (46.96%)	90 (42.86%)	
Lumpectomy	216 (55.24%)	96 (53.04%)	120 (57.14%)	
Unknown/missing	163	92	71	
Body mass index				0.239
<25 kg/m2	174 (33.46%)	77 (29.96%)	97 (36.88%)	
25 to < 30 kg/m^2^	133 (25.58%)	68 (26.46%)	65 (24.71%)	
≥30 kg/m^2^	213 (40.96%)	112 (43.58%)	101 (38.4%)	
Unknown/missing	34	16	18	

*Note*: The values are presented as Mean ± standard deviation for continuous variables and *n* (%) for categorical variables. The unknown/missing cases were not included in the percentage calculation.

Abbreviations: AET, adjuvant endocrine therapy; HER2, human epidermal growth factor receptor 2.

^a^ Number of non‐consecutive valid data points out of 4 years (baseline, Year 1 through 3).

^b^ *p*‐values are estimated from an independent sample *t*‐test for a continuous variable and *x* ^2^ test of independence for a categorical variable.

^c^ Those who reported working full‐ and part‐time were categorized as “employed”. Otherwise (e.g., full‐time homemaker, retired, student) were categorized as “unemployed.”

^d^ women underwent both lumpectomy and mastectomy (*n* = 16) were categorized as the mastectomy group.

Descriptive statistics of the primary outcomes of interest are shown in Table S1. At baseline women reported engaging in an average of 16.94 ± 15.33 MET‐hours of PA per week; this increased significantly over the first year to 26.9 MET‐hours/week, and then to almost double the baseline level by the year 3 follow‐up (30.54 ± 19.91, *p* < 0.001). In contrast, time spent sitting did not change over time and remained between 6 and 7 h per day. Baseline QOL and endocrine symptoms scores (FACT‐G; 87.55 ± 12.59, FACT‐B; 115.82 ± 16.60, FACT‐ES subscale; 15.72 ± 9.99) all got significantly worse by an average of 1–3 points over time (*ps* < 0.01). Table 2 shows the results of the unconditional LGCM's examining the estimated change in each outcome of interest across the 3 years of follow‐up. Latent growth parameters estimated from each model showed that PA increased significantly (*Slope*) by approximately 4.7 MET‐hours per week each year while sitting time did not change significantly over time. General and BC specific QOL worsened by an estimated 1.3 and 1.9 points per year while endocrine symptoms increased by 1.5 points per year.

**TABLE 2:** Unconditional latent growth curve modeling on changes in study outcomes over a 4‐year period (*n* = 554).

	Model‐data fit indices				Latent growth factors		
	*x* ^2^ (*df*)	CFI	TLI	RMSEA	Intercept (SE)^a^	Slope (SE)^a^	Correlation (SE)^b^
Total MET‐hours/week	34.35 (5)**	0.809	0.781	0.103	17.90 (0.66)**	4.66 (0.46)**	0.23 (0.39)
Sitting time (h/day)^c^	4.92 (4)	0.987	0.980	0.021	6.83 (0.15)**	−0.19 (0.13)	−0.01 (0.40)
FACT‐ES (subscale only)	38.89 (5)**	0.949	0.939	0.111	16.27 (0.42)**	1.45 (0.17)**	0.32 (0.34)
FACT‐general	28.20 (5)**	0.956	0.947	0.092	87.37 (0.54)**	−1.29 (0.30)**	0.16 (0.16)
FACT‐breast	35.99 (5)**	0.947	0.936	0.106	115.53 (0.71)**	−1.88 (0.39)**	0.26 (0.16)

Abbreviations: CFI, comparative fit index; ES, Endocrine symptoms; FACT‐Breast, Functional Assessment of Cancer Therapy‐Breast Cancer (FACT‐G + Breast cancer subscale); FACT‐General, Functional Assessment of Cancer Therapy‐General (Physical + Social/Family + Emotional + Functional); MET, metabolic equivalent tasks; RMSEA, root mean square error of approximation; TLI, Tucker–Lewis index.

^a^ The values are the estimated mean growth factors (standard error).

^b^ The correlation between the intercept and slope.

^c^ The residual covariance between the Year 1 and 3 was added in the model to improve the model‐data‐fit.

* *p* < 0.05.

** *p* < 0.001.

### 3.2 Physical activity, quality of life, and endocrine symptoms

Table 3 presents the results of the parallel LGCM examining the associations between PA, endocrine symptoms and QOL over time. The results showed there was no significant association between baseline PA and endocrine symptoms. A significant positive cross‐sectional association was found between PA and both general (*b* = 0.25; *p* = 0.023) and BC‐specific (*b* = 0.29; *p* = 0.011) QOL at baseline (*Intercept‐to‐Intercept correlations)*. Prospective associations between baseline levels of PA and changes in endocrine symptoms and QOL (*Intercept*_*1*_ *‐to‐Slope*_*2*_) were not statistically significant. The reverse was also true whereby baseline levels of endocrine symptoms and QOL were not significantly associated with a change in PA over time (*Intercept*_*2*_ *‐ to‐Slope*_*1*_). Similarly, changes in PA were not significantly associated with changes in endocrine symptoms or QOL (*Slope*_*1*_ *‐ to‐Slope*_*2*_ *)*.

**TABLE 3:** The results of parallel process latent growth model between total MET‐hour/week and endocrine symptoms and QOL (FACT).

	FACT‐ES^a^		FACT‐General		FACT‐Breast	
	*b* (SE)	*p*	*b* (SE)	*p*	*b* (SE)	*p*
Intercept‐to‐intercept correlation						
(a) I_1_↔I_2_	−0.10 (0.08)	0.206	0.25 (0.11)	**0.023**	0.29 (0.11)	**0.011**
Intercept‐to‐slope coefficients						
(b) I_1_→S_2_	0.41 (0.27)	0.132	−0.19 (0.24)	0.442	−0.10 (0.20)	0.477
(c) I_2_→S_1_	−0.08 (0.05)	0.133	0.24 (0.13)	0.061	0.08 (0.04)	0.071
Slope‐to‐slope coefficient						
(d) S_1_→S_2_	−0.65 (0.43)	0.132	0.31 (0.23)	0.188	0.23 (0.22)	0.299
Model‐data fit indices						
*x* ^2^ (df)	93.30 (36)**		92.06 (34)**		92.28 (34)**	
CFI	0.928		0.916		0.921	
TLI	0.896		0.872		0.879	
RMSEA	0.058		0.060		0.060	

Abbreviations: CFI, comparative fit index; FACT‐ES, Functional Assessment of Cancer Therapy–Endocrine Symptoms (subscale only); I, intercept of the latent growth factors; the models were adjusted for age, race, and time since AET initiation; I_1_, intercept growth factor for total MET‐hours/week; I_2_, intercept growth factor for FACT‐ES subscale scores; RMSEA, root mean square error of approximation; S, slope of the latent growth factors; S_1_, slope growth factor for total MET‐hours/week; S_2_, slope growth factor for FACT‐ES subscale scores; TLI, Tucker‐Lewis index.

^a^ The residual variance of slope (S_2_) was fixed to zero due to the negative value.

* *p* < 0.05.

** *p* < 0.001.

### 3.3 Sitting time, quality of life, and endocrine symptoms

Table 4 presents the results of the parallel LGCM examining the associations between sitting, endocrine symptoms and QOL over time. There was a significant association between baseline sitting and endocrine symptoms with more sitting time associated with worse endocrine symptoms (*b* = 0.27; *p* = 0.008). A significant cross‐sectional association was also found between sitting and general (*b* = −0.52; *p* < 0.004) and BC‐specific (*b* = −0.50; *p* < 0.001) QOL (*Intercept‐to‐Intercept correlations)* showing that more sitting was associated with having worse QOL scores. Prospective associations between baseline levels of sitting and changes in endocrine symptoms or QOL were not significant (*Intercept*_*1*_ *‐to‐Slope*_*2*_). However, the reverse associations (*Intercept*_*2*_ *‐to‐Slope*_*1*_) were significant; worse endocrine symptoms at baseline were associated with a slower rate of increase in sitting time (*b* = −0.04; *p* = 0.031), and better general (*b* = 0.04; *p* = 0.030) and BC‐specific (*b* = 0.04; *p* = 0.003) QOL scores at baseline were associated with a slightly increased rate of sitting over time. Slope to slope associations showed that an increase in sitting over time was significantly associated with lower rate of increase in endocrine symptoms (*b* = −2.61; *p* = 0.046), and while also being associated with a decline in general and BC‐specific QOL (*Slope*_*1*_ *‐ to‐Slope*_*2*_); those associations were not significant.

**TABLE 4:** The results of parallel process latent growth model between sitting time (min/day) and endocrine symptoms and QOL (FACT).

	FACT‐ES^a^ ^,^ ^b^		FACT‐General^a^		FACT‐Breast^a^	
	*b* (SE)	*p*	*b* (SE)	*p*	*b* (SE)	*p*
Intercept‐to‐intercept correlation						
(a) I_1_↔I_2_	0.27 (0.10)	**0.008**	−0.52 (0.18)	**0.004**	−0.50 (0.13)	**<0.001**
Intercept‐to‐slope coefficients						
(b) I_1_→S_2_	−0.32 (0.17)	0.069	1.90 (7.57)	0.802	−0.39 (0.86)	0.654
(c) I_2_→S_1_	−0.04 (0.02)	**0.031**	0.04 (0.02)	**0.030**	0.04 (0.01)	**0.003**
Slope‐to‐slope coefficient						
(d) S_1_→S_2_	−2.61 (1.31)	**0.046**	−6.82 (22.19)	0.758	−2.67 (2.03)	0.189
Model‐data fit indices						
*x* ^2^ (df)	61.54 (37)**		51.92 (33)		68.16 (29)	
CFI	0.965		0.968		0.944	
TLI	0.950		0.949		0.915	
RMSEA	0.037		0.035		0.049	

*Note*: The schematic diagram of the parallel process model is depicted in Figure 1. The models were adjusted for age, race, and time since AET initiation for FACT‐ES and FACT‐General. However, for FACT‐ES, the models were adjusted for age and race only, due to a model convergence issue with the time since AET initiation.

Abbreviations: CFI, comparative fit index; FACT‐ES, Functional Assessment of Cancer Therapy–Endocrine Symptoms (subscale only); I, intercept of the latent growth factors; I_1_, intercept growth factor for sitting time (hours/day); I_2_, intercept growth factor for FACT‐ES subscale scores; IPAQ, International Physical Activity Questionnaire; RMSEA, root mean square error of approximation; S, slope of the latent growth factors; S_1_, slope growth factor for sitting time (hours/day); S_2_, slope growth factor for FACT‐ES subscale scores; TLI, Tucker‐Lewis index.

^a^ The residual covariance between the year 1 and 3 was added in the model to improve the model‐data‐fit.

^b^ The residual variances of slope (S_1_ and S_2_) were fixed to zero due to the negative values.

* *p* < 0.05.

** *p* < 0.001.

## 4 DISCUSSION

In this secondary analysis of the WHIP Study examining cross‐sectional and longitudinal associations among weekly PA, daily sitting time, endocrine symptoms and QOL, we found significant associations between activity levels (and sitting time) with endocrine symptoms and general and BC specific QOL. Self‐reported PA increased each year, while sitting time stayed constant. Endocrine symptoms significantly increased (worsened) while general and BC specific health‐related QOL declined slightly over the same period, with the majority of change apparently in the first year of the study. Importantly, higher levels of baseline PA or change in PA were not associated with changes in endocrine symptoms or health‐related QOL over time. We did however find that worse endocrine symptoms were associated with a slower rate of increase in sitting time while having better QOL scores at baseline was associated with a significant increase in the rate of sitting. Overall, both PA and sitting appear to be important behaviors among BC survivors undergoing AET.

A number of previous studies examining the relationship between PA, endocrine symptoms and QOL among women with BC have predominantly examined arthralgias or musculoskeletal concerns that develop as a result of aromatase inhibitor (AI) therapy for HR+ BC. One recent meta‐analysis comparing exercise or usual care, examined nine trials with 743 participants^23^ and found exercise to be an effective approach for managing pain, stiffness and grip strength. However, in separate smaller studies^19^, ^20^ BC patients did not experience improvements in QOL domains such as fatigue, endocrine symptoms or total quality of life, despite the significant improvements in PA behaviors as a result of intervention. While not all symptoms that result from AET are musculoskeletal in nature, their presence is also linked to reductions in PA among women who take them.^34^ Other factors linked to declines of PA following use of AIs included BMI. In the HOPE study,^21^ a 12‐month clinical exercise trial among women with AI‐induced arthralgia, 121 BC survivors with at least mild arthralgias were randomized to a supervised exercise or control group. At 12 months follow‐up, women who participated in aerobic and resistance training reported greater improvements in overall, BC‐specific and endocrine symptom subscales compared to the control participants. Study authors concluded that given the frequency of side effects from AIs and the risk for treatment non‐adherence, that non‐pharmacological approaches like exercise training could be valuable. While our results found higher baseline levels of activity to be associated with better QOL, we did not see an improvement in QOL (general or BC‐specific) or endocrine symptoms to be associated with PA. This may be due to the fact that baseline activity was already relatively high with the average self‐reported activity levels being above the level of the current PA guidelines. Furthermore, in our study walking activity was likely the most common activity reported due to the non‐specific nature of the IPAQ regarding resistance training, which was a primary component of the exercise intervention in the HOPE study. It should be noted that in our study we only assessed general quality of life (FACT‐G and FACT‐B) and did not specifically examine domain specific changes that may be associated with PA or sitting. For example, Baglia et al.,^21^ both aerobic and resistance exercise training effects on QOL over 12 months, reported improvements in both physical and functional well‐being as the primary driver of change in general QOL captured by the FACT and also found endocrine symptom specific improvements. It is well established that resistance training is critical for the maintenance of physical functioning, including among older persons and those who experience chronic pain. Therefore, guidance to increase physical activity should specifically include the guideline‐based recommendations of at least 2 days of resistance training per week in addition to at least 150 min of aerobic training.

A unique aspect and strength of this study is the examination of sitting time and its association with QOL and endocrine symptoms cross‐sectionally and over time. Our finding that worse endocrine symptoms and better QOL baseline, were associated with slower and faster rates of increase in sitting over time, respectively, was somewhat unexpected. Women with better QOL at baseline may have increased their sitting as they began to experience side effects of treatment. Women who experience pain at either at rest or at onset of activity, are likely to attribute this discomfort to activity and therefore do less. Unfortunately, this only serves to further reduce their functional capacity increasing the likelihood that when they engage in activity, they will experience discomfort. Similarly, it is often counter‐intuitive that women who are fatigued can benefit from activity. Most often patients who are fatigued believe they should do less and rest, which again leads to decline in function and worsening fatigue over time. The only way to combat this is to engage in activity to strengthen the musculoskeletal system. Indeed, our prior findings indicated that women who were the most adherent to AET had the highest levels of sitting time,^35^ perhaps suggesting that these women would experience side effects of AET early and these symptoms would then level off or increase more slowly over time. Two recent studies, examining both physical activity and sedentary behavior using accelerometry among post‐menopausal BC survivors or post‐systemic therapy BC survivors (50% on hormonal therapy),^36^, ^37^ found that more time spent sitting, and in longer bouts was associated with worse physical QOL, especially in women who did little activity. While we did not specifically assess whether there was effect modification in our analyses, it may be that women who were already doing more PA were also resting more outside of those bouts of activity. These findings highlight that both behaviors are independently important for the management of symptoms in BC survivors and they should be advised to both increase exercise and also reduce long periods of sitting time. Other strengths of our study are the inclusion of a racially diverse sample of HR+ BC survivors.

This study does have a few limitations. First, only self‐reported measures were used for collecting PA and sitting data. While important for clarifying context, there are known issues with over‐reporting of PA and under‐reporting of sitting time in the IPAQ questionnaire, particularly for BC survivors.^38^, ^39^, ^40^ Furthermore, use of objective measures of PA would allow for the assessment of lower intensities of PA in the range of 1.5–2.9 METs, capturing movement and daily activities of living which are lost in self‐reported measures but still important for health among BC survivors.^16^ Fortunately, because the primary outcome of interest in the WHIP parent study was adherence to AET and not specifically PA there may have been less inclination to overreport activity. Nevertheless, the use of accelerometers would have improved the accuracy of data capture in that regard. Second, there were a relatively large number of participants who were lost to follow‐up in this study. We did find that certain baseline characteristics (race, education, and income) seemed to be correlated with missingness of study variables; however, in sensitivity analyses the primary findings were not altered after excluding those with two or more missing visits. Thus, we cannot fully rule out the possibility of survival bias where the missingness may be related to the symptoms or prognosis after treatment. Further, we only included the treatment type at the time of the baseline visit in these models and did not explore whether women changed the type of therapy used over time such as in a switch strategy. This implies that caution is needed, particularly when interpreting the results from an unconditional LGCM showing the shape of longitudinal changes in study variables over time. Lastly, the model‐data fits of the LGCMs were below or marginally above the acceptable levels for model fit parameters in structural equation modeling frameworks. The greater model‐data fits are necessary to obtain the valid parameter estimates from the LGCM model, and thus future studies are warranted to test the proposed LGCM model in different settings.

In summary, our findings from a large and diverse cohort of BC survivors undergoing AET, shows that both PA and sitting time were important with respect to managing symptoms and maintaining QOL. Clinician guidance towards the adoption of PA that includes both aerobic and resistance exercise and in addition, a reduction of sedentary behaviors should take into account the patient‐level variations in symptoms such as fatigue or pain, that may make activity challenging. Regular activity can also help with weight management efforts, which is important for a population of women who are at risk for weight gain and therefore further comorbid conditions. Given baseline levels of activity/sitting are more strongly associated with baseline symptoms and QOL, it is important that women are encouraged to adopt appropriate activity behaviors as early as possible, despite the challenges they may face as a result of treatment and associated side effects. Future research is needed to more accurately delineate the optimal prescription of PA (e.g., type, dose, intensity, volume) coupled with management of prolonged periods of inactivity (sedentary behaviors), especially in consideration of the types of treatment a women may receive for HER+ BC.

## AUTHOR CONTRIBUTIONS

**Alexander R. Lucas:** Conceptualization (lead); data curation (equal); formal analysis (equal); methodology (lead); writing – original draft (lead); writing – review and editing (equal). **Youngdeok Kim:** Data curation (equal); formal analysis (lead); methodology (equal); writing – original draft (supporting); writing – review and editing (equal). **Autumn Lanoye:** Writing – original draft (supporting); writing – review and editing (equal). **R. Lee Franco:** Writing – original draft (supporting); writing – review and editing (equal). **Arnethea L. Sutton:** Writing – original draft (supporting); writing – review and editing (equal). **Jessica Gokee LaRose:** Writing – original draft (supporting); writing – review and editing (equal). **Masey Ross:** Writing – original draft (supporting); writing – review and editing (equal). **Vanessa B. Sheppard:** Writing – original draft (equal); writing – review and editing (equal).

## FUNDING INFORMATION

This research was funded by the National Institutes of Health (R01CA154848) and the Bank of America (to Vanessa B. Sheppard). It was also supported in part by the National Cancer Institute (2T32CA093423 to Vanessa B. Sheppard), the National Institutes of Health/National Cancer Institute (Cancer Center Support Grant P30 CA016059), and the National Center for Advancing Translational Sciences (Translational Science Award UL1TR002649). Its contents are solely the responsibility of the authors and do not necessarily represent official views of the National Center for Advancing Translational Sciences or the National Institutes of Health.

## CONFLICT OF INTEREST STATEMENT

The authors have no relevant financial or non‐financial interests to disclose.

## ETHICS STATEMENT

All study procedures were approved by Institutional Review Boards at participating sites across 2 primary geographic regions (Midwest, Southeast) from academic medical centers, from integrated health systems, and via community outreach efforts.

## PATIENT CONSENT STATEMENT

All participants interested in participating in this study provided informed consent via an approved telephone screening process.

## Supporting information


Table S1.

## Data Availability

The datasets generated during and/or analyzed during the current study are available from the corresponding author on reasonable request.
