# Supplementary material for: Longitudinal associations among physical activity and sitting with endocrine symptoms and quality of life in breast cancer survivors: A latent growth curve analysis
Source: Cancer Med. 2023 Sep 28;12(19):20094–105. doi: 10.1002/cam4.6581 (PMC10587924; doi:10.1002/cam4.6581)
Supplement: Supplementary file 1 — Table S1. [file CAM4-12-20094-s001.docx]

### Supplemental Table1. Descriptive Statistics of Physical Activity Levels, Sitting and Endocrine Symptoms and QOL (FACT) Over Time

|  | Baseline | | Year 1 | | Year 2 | | Year 3 | | *P*-for-trends^a^ |
| --- | --- | --- | --- | --- | --- | --- | --- | --- | --- |
|  | *n* | *Mean*±*SD* | *n* | *Mean*±*SD* | *n* | *Mean*±*SD* | *n* | *Mean*±*SD* |  |
| Total MET-hours/wk | 525 | 16.94±15.33 | 363 | 26.90±19.26 | 308 | 27.76±19.34 | 142 | 30.54±19.91 | **<.001** |
| Sitting time (hours/day) | 508 | 6.89±3.64 | 337 | 6.37±4.43 | 294 | 6.32±4.98 | 134 | 6.98±7.23 | .901 |
| FACT-ES (subscale only) | 526 | 15.72±9.99 | 366 | 18.78±9.77 | 313 | 18.73±9.67 | 145 | 17.97±9.66 | **<.001** |
| FACT-General | 527 | 87.55±12.59 | 368 | 85.96±14.94 | 314 | 85.71±14.66 | 145 | 86.51±14.18 | **.005** |
| FACT-Breast | 527 | 115.82±16.60 | 368 | 113.53±19.73 | 314 | 113.19±19.68 | 145 | 114.89±18.15 | **.003** |

ES = Endocrine symptoms; FACT-General = Functional Assessment of Cancer Therapy-General (Physical + Social/Family + Emotional + Functional); FACT-Breast = Functional Assessment of Cancer Therapy-Breast Cancer (FACT-G + Breast cancer subscale); MET = metabolic equivalent tasks

^a^ Linear trends were estimated using a random intercept mixed model.
